# The Tat system and its dependent cell division proteins are critical for virulence of extra-intestinal pathogenic *Escherichia coli*

**DOI:** 10.1080/21505594.2020.1817709

**Published:** 2020-09-22

**Authors:** Jinjin Liu, Fan Yin, Te Liu, Shaowen Li, Chen Tan, Lu Li, Rui Zhou, Qi Huang

**Affiliations:** a State Key Laboratory of Agricultural Microbiology, College of Veterinary Medicine, Huazhong Agricultural University, Wuhan, China; b Cooperative Innovation Center for Sustainable Pig Production, College of Veterinary Medicine, Huazhong Agricultural University, Wuhan, China; c International Research Center for Animal Disease, Ministry of Science and Technology, Wuhan, China; d Key Laboratory of Development of Veterinary Diagnostic Products, Ministry of Agriculture and Rural Affairs of China, Wuhan, China

**Keywords:** Twin-arginine protein translocation (Tat) system, extra-intestinal pathogenic *Escherichia coli* (ExPEC), Tat substrate protein, cell division, stress response, pathogenesis

## Abstract

The twin-arginine translocation (Tat) system is involved in a variety of important bacterial physiological processes. Conserved among bacteria and crucial for virulence, the Tat system is deemed as a promising anti-microbial drug target. However, the mechanism of how the Tat system functions in bacterial pathogenesis has not been fully understood. In this study, we showed that the Tat system was critical for the virulence of an extra-intestinal pathogenic *E. coli* (ExPEC) strain PCN033. A total of 20 Tat-related mutant strains were constructed, and competitive infection assays were performed to evaluate the relative virulence of these mutants. The results demonstrated that several Tat substrate mutants, including the Δ*sufI*, Δ*amiA*Δ*amiC* double mutant as well as each single mutant, Δ*yahJ*, Δ*cueO*, and Δ*napG*, were significantly outcompeted by the WT strain, among which the Δ*sufI* and Δ*amiA*Δ*amiC* strains showed the lowest competitive index (CI) value. Results of individual mouse infection assay, *in vitro* cell adhesion assay, whole blood bactericidal assay, and serum bactericidal assay further confirmed the virulence attenuation phenotype of the Δ*sufI* and Δ*amiA*Δ*amiC* strains. Moreover, the two mutants displayed chained morphology in the log phase resembling the Δ*tat* and were defective in stress response. Our results suggest that the Tat system and its dependent cell division proteins SufI, AmiA, and AmiC play critical roles during ExPEC pathogenesis.

## Introduction

Protein translocation and secretion are critical for bacterial survival, environmental adaption, and pathogenesis [1,2]. So far, a variety of protein secretion systems have been discovered in bacteria, among which the twin-arginine translocation (Tat) system is a unique one that facilitates folded proteins to be inserted into or translocated across the cytoplasmic membrane [2–5]. The Tat system is present in most bacteria, plant chloroplasts, but absent in mammalian cells. It is composed of a core membrane protein TatC and one or two TatA-like proteins [6]. The proteins exported through the Tat pathway encompass an SRRxFLK motif at their N-terminus [7]. In *E. coli*, over 30 proteins have been verified or predicted to be transported through the Tat pathway, which are distributed in diverse physiological pathways [5,8].

Due to the diversity of Tat substrate proteins, Tat system disruption causes pleiotropic defects, affecting bacterial growth, cell division, motility, biofilm formation, iron acquisition, stress response, *etc* [9,10]. Moreover, the crucial role of the Tat system in pathogenesis has been reported in several important bacterial pathogens, including *Pseudomonas aeruginosa* [11], *E. coli* O157 [12], *Salmonella enterica* serovar Typhimurium [13,14], *Citrobacter freundii* [15], and *Yersinia pseudotuberculosis* [16]. Efforts have been made by several research groups to unravel how the Tat system affects bacterial virulence. Envelop defects are previously proposed to be the major cause of virulence attenuation in *S*. Typhimurium [17]. However, a recent study shows that it is the cell division defects under stress conditions in the gut that contribute to the *in vivo* fitness decrease of the *tat* mutant of *S*. Typhimurium [18]. Besides, the Tat system has been suggested to affect the efﬁciency of type III secretion in a plant pathogen *P. syringae* [19]. However, it is not the case in *S*. Typhimurium [17]. Therefore, the role of the Tat system during bacterial pathogenesis needs further investigation.

Extra-intestinal pathogenic *Escherichia coli* (ExPEC) is one of the most important pathogens causing neonatal meningitis, urinary tract infections, and sepsis in humans [20]. Recently, ExPEC has also been frequently isolated from livestock which are believed as important reservoirs causing human infections [21–23]. A substantial amount of ExPEC isolates of animal origin were reported that possessed high-level antimicrobial resistance and were highly virulent, and some isolates share similar virulence factors with those of human origin [24–26]. Therefore, ExPEC is becoming a potential threat to food safety as well as public health. Understanding the pathogenesis of the ExPEC is of great significance.

In this study, we reveal that the Tat system is critical for the virulence of ExPEC. By constructing Tat-related mutants and performing competitive infection assays, we showed that the Tat-dependent cell division proteins SufI, AmiA, and AmiC are the key Tat substrate proteins accounting for the virulence attenuation of the *tat* mutant. Further analysis suggests that Δ*sufI* and Δ*amiA*Δ*amiC* displayed severe cell division defect in the log phase and their growth was compromised under different stress conditions.

## Materials and methods

### Bacterial strains and cell culture conditions

All strains used in this study are listed in Table 1. The ExPEC strain PCN033 was isolated from the brain of a diseased pig with meningitis as described previously [27,28], which exhibited meningitis and high virulence in the mouse infection model [29,30]. *E. coli* strain χ7213 is a diaminopimelic acid (DAP) autotrophic strain used for delivering plasmid into ExPEC PCN033 through transconjugation [31]. *E. coli* DH5α λ*pir* was used as the host strain for the propagation of pRE112 [32] or its derived plasmids. *E. coli* DH5α was used as the host strain for routine cloning. The *E. coli* χ7213 is grown in LB supplemented with 50 μg/mL of DAP. Chloramphenicol and apramycin were used at a final concentration of 50 μg/ml. PK-15 (pig kidney epithelial cell) and BHK-21 (derived from baby hamster kidney) cells were cultured in Dulbecco’s modified Eagle’s medium (DMEM) (Invitrogen, Carlsbad, CA, USA) supplemented with 10% heat-inactivated fetal bovine serum in a 37°C incubator with 5% CO_2_.

**Table 1.** Strains used in this study.

Strain	Description	Source
*E. coli* PCN033	Wild type ExPEC strain, highly virulent, isolated from pig brain	^[27]^
*E. coli* DH5α	Cloning host strain	Vazyme Biotech Co., Ltd.
*E. coli* DH5α λ*pir*	Cloning host strain	^[33]^
*E. coli χ*7213	Diaminopimelic acid autotrophic strain used in transconjugation.	^[31]^
*Δtat*	As *E. coli* PCN033, *tatABC* deleted.	This work
*Δtat-Cm*	As *E. coli* PCN033, *tatABC* replaced with chloramphenicol resistance cassette. Cm^R^.	This work
Δ*amiA*	As *E. coli* PCN033, *amiA* replaced with chloramphenicol resistance cassette. Cm^R^.	This work
Δ*amiA2*	As *E. coli* PCN033, in-frame deletion of *amiA*.	This work
Δ*amiC*	As *E. coli* PCN033, *amiC* replaced with chloramphenicol resistance cassette. Cm^R^.	This work
Δ*amiA*Δ*amiC*	As Δ*amiA2, amiC* replaced with chloramphenicol resistance cassette. Cm^R^.	This work
Δ*sufI*	As *E. coli* PCN033, *sufI* replaced with chloramphenicol resistance cassette. Cm^R^.	This work
Δ*moaA*	As *E. coli* PCN033, *moaA* replaced with chloramphenicol resistance cassette. Cm^R^.	This work
Δ*cueO*	As *E. coli* PCN033, *cueO* replaced with chloramphenicol resistance cassette. Cm^R^.	This work
Δ*yahJ*	As *E. coli* PCN033, *yahJ* replaced with chloramphenicol resistance cassette. Cm^R^.	This work
Δ*wcaM*	As *E. coli* PCN033, *wcaM* replaced with chloramphenicol resistance cassette. Cm^R^.	This work
Δ*modD*	As *E. coli* PCN033, *modD* replaced with chloramphenicol resistance cassette. Cm^R^.	This work
Δ*fhuD*	As *E. coli* PCN033, *fhuD* replaced with chloramphenicol resistance cassette. Cm^R^.	This work
Δ*ycbK*	As *E. coli* PCN033, ycbK replaced with chloramphenicol resistance cassette. Cm^R^.	This work
Δ*efeOB*	As *E. coli* PCN033, *efeOB* replaced with chloramphenicol resistance cassette. Cm^R^.	This work
Δ*fdnG*	As *E. coli* PCN033, *fdnG* replaced with chloramphenicol resistance cassette. Cm^R^.	This work
Δ*fdoG*	As *E. coli* PCN033, *fdoG* replaced with chloramphenicol resistance cassette. Cm^R^.	This work
Δ*hyaA*	As *E. coli* PCN033, *hyaA* coding sequence replaced with chloramphenicol resistance gene coding sequence.	This work
Δ*napG*	As *E. coli* PCN033, *napG* replaced with chloramphenicol resistance cassette. Cm^R^.	This work
Δ*hybAO*	As *E. coli* PCN033, *hybAO* replaced with chloramphenicol resistance cassette. Cm^R^.	This work
Δ*nrfC*	As *E. coli* PCN033, *nrfC* coding sequence replaced with chloramphenicol resistance gene coding sequence.	This work
Δ*yagT*	As *E. coli* PCN033, *yagT* coding sequence replaced with chloramphenicol resistance gene coding sequence.	This work
Δ*ydhX*	As *E. coli* PCN033, *ydhX* coding sequence replaced with chloramphenicol resistance gene coding sequence.	This work
CΔ*tat*	*ΔtatABC* transformed with pHSG396-*tatABC*	This work
CΔ*sufI*	Δ*sufI* transformed with pHSG396Apra-*sufI*	This work

### Construction of plasmids and mutant strains

All primers and plasmids used in this study are listed in Table S1 and Table S2, respectively. Plasmids were constructed by seamless cloning using the ClonExpress® MultiS One Step Cloning Kit (Cat# C113, Vazyme Biotech Co., Ltd, Nanjing, China). All the mutant strains were constructed as described previously [30]. Briefly, *E. coli* χ7213 competent cells were transformed with a pRE112 derived plasmid, which served as the donor strain for transconjugation. Cells of donor strain and recipient strain were mixed with a ratio of 10:1 and dripped onto a sterile filter membrane disc (Φ 0.45 μm) which was then placed on LB agar plate containing 50 μg/ml of DAP followed by incubation at 37°C for 5 hours. The bacterial cells were washed off from the disc and the cells plated onto LB agar containing chloramphenicol followed by overnight growth at 37°C. The colonies were picked and single exchanged strains were identified by PCR. The double exchanged mutants were then screened by using LB agar plate containing 10% sucrose and PCR identification of the presence of the target gene.

### Growth assay

To measure bacterial growth in liquid medium, overnight-grown cell culture was diluted in LB medium giving an initial OD_600nm_ of 0.01 and grown at 37°C with shaking at 200 round/min (rpm). OD_600nm_ was recorded at each time point using a spectrometer. When necessary, cell culture at certain time point was taken, diluted in LB, and plated onto LB plate for viable cell counting. When doing growth assay on agar plates, bacterial cells at the mid-log phase were diluted in LB to give an identical OD_600nm_ value, which were subject to 10-fold dilution, and 3 μL of cells of each indicated strain were spotted onto each specific LB agar plate. When measuring bacterial resistance against porcine β-defensin 2 (PDB2), 5 × 10^3^ CFU of bacterial cells were mixed with different concentrations of synthetic porcine β defensin at 37°C for 1 hour, and the samples were plated onto LB plate and the viable cells were counted. The plates were then grown at each indicated temperature. The assay was performed in triplicate.

### Morphological analysis

Gram staining and fluorescence imaging were used to analyze bacterial morphology. For Gram staining, cells of each strain were grown in LB to the log phase, washed three times with PBS, stained with Gram staining reagents according to the regular procedure, and observed with an optical microscope. For fluorescence imaging, each strain was transformed with pQE80Apra-GFP plasmid which constitutively expresses green fluorescent protein. The cells were grown in LB and harvested at each indicated time point. The cells were washed three times with PBS and imaged using a fluorescence confocal microscope.

### Bacterial swimming assay

The swimming assay was performed as previously described [18]. Briefly, 5 μL of cells of each indicated strain at the mid-log phase (OD_600nm_ at 0.6) were spotted onto agar plate containing 10 g/L tryptone, 5 g/L yeast extract, 5 g/L NaCl, 0.5% glucose (w/v), and 0.45% agar (w/v). The plate was photographed after incubation at 37°C for 8 h.

## Animal infection experiments

All animal experiments were approved by the Laboratory Animal Monitoring Committee of Huazhong Agricultural University and performed according to the recommendations in the Guide for the Care and Use of Laboratory Animals of Hubei Province, China. Female Kunming mice were purchased from the Experimental Animal Center, Huazhong Agricultural University, Wuhan, China. When evaluating the virulence of an individual ExPEC strain, bacterial cells were grown to the mid-log phase, pelleted, washed with sterile saline, and diluted in sterile saline to get an appropriate amount of viable cells which were then used to inject mouse intraperitoneally. The survival rate of the mice was recorded every 24 hours post-infection for 7 days. When calculating the *in vivo* bacterial loads, the mice were euthanized at each indicated time points and the organs were collected, weighed, homogenized in sterile saline, and plated on to LB for cell counting. Competitive infection assay, an accurate and sensitive approach to determine relative virulence, was used to determine whether the mutant was attenuated compared with the WT strain [17,34–36]. In the competitive infection assay, a similar amount of viable cells in the mid-log phase of the WT strain and each Tat-related mutant were mixed and used to inject mouse intraperitoneally. The mice were euthanized at each indicated time point, and the number of the WT strain and mutant strain in each organ were counted after plating on LB plate with or without chloramphenicol, respectively. The competitive index (CI) was calculated by dividing the ratio of the mutant cells to the WT cells recovered from the tissues by the ratio of the mutant cells to the WT cells in the injection mixture [output (CFU_mutant_/CFU_WT_)/Input (CFU_mutant_/CFU_WT_)]. When *yagT, nrfC, hyaA* and *ydhX*, which were located within operons, were in-frame substituted with Cm^r^ coding sequence, the strains did not show chloramphenicol resistance. To calculate the competitive index for these strains, the numbers of the WT strain and the mutant strain in the recovered colonies were determined by using PCR.

### In vitro *cell adhesion assay, whole blood bactericidal assay, and serum bactericidal assay*

PK-15 and BHK-21 cells were used to test the adhesion of the WT and the mutant strains as previously described [30]. Briefly, bacterial cells grown to mid-log phase were harvested and washed three times with DMEM. PK-15 cells and BHK-21 cells grown in 6-well plates were infected with cells of each indicated bacterial strain with a ratio of 10:1 followed by incubation at 37°C with 5% CO_2_ for 2 hours. The cells were then washed five times with PBS and lysed with sterile water. The input bacterial cells and the cell lysates were then diluted and plated onto LB plates for bacterial enumeration. The adhesion rate of the WT strain was set as 100%. Whole blood bactericidal assay was performed as previously described [37]. Briefly, 450 μL heparinized mouse whole blood were mixed with 50 μL bacterial cells of each indicated strain grown to mid-log phase (approximately 10^8^ CFU/mL) and incubated at 37°C for 1 hour. The samples were then diluted and plated onto LB plates for bacterial enumeration. Serum bactericidal assay was performed as previously described [30,38]. Briefly, 100 μL bacterial cells of each indicated strain grown to mid-log phase (approximately 10^8^ CFU/mL) were mixed with 100 μL of normal mouse serum (NS), or serum inactivated at 56°C for 30 min (IS) at 37°C, for 1 hour. The initial input samples and the incubated samples were then diluted and plated onto LB plates for bacterial enumeration. The survival rate was calculated as (CFU_recovered_/CFU_input_) × 100.

### Statistical analysis

Statistical analysis was performed using GraphPad Prism (version 5) software. The Student’s t-test was used to calculate the differences between two groups. Error bars in the graphs represent the standard deviations of the means.

## Results

### The Tat system is essential for the virulence of ExPEC

**Figure 1.**
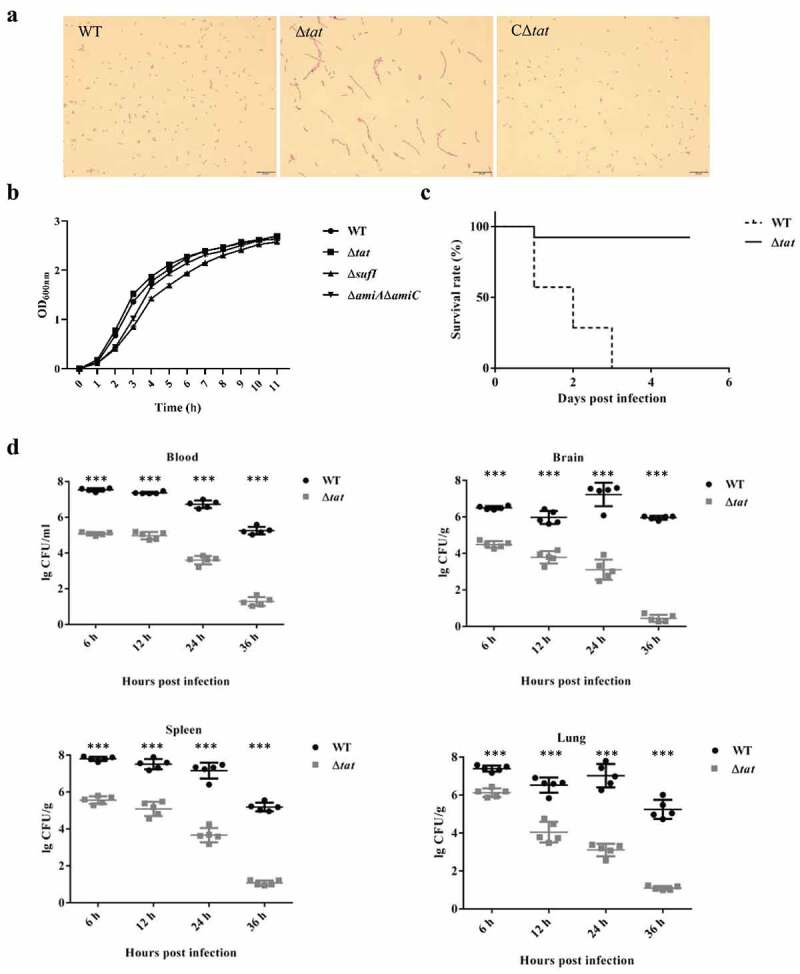
Morphological, growth and virulence characterization of the parental ExPEC PCN033 strain and the *tat* mutant (Δ*tat*).

In the genome of ExPEC strain PCN033, an operon encoding TatA, TatB and TatC was present. To test whether the Tat system was functional, we constructed a *tatABC* deletion mutant (Δ*tat*) and its complement strain (CΔ*tat*). As shown in Figure 1a, the Δ*tat* formed chained morphology in contrast to the wild-type (WT) and the complement strains, which was consistent with the observation reported in previous studies [39,40]. This suggested that the Tat system was functional in the ExPEC PCN033 strain. The growth assay showed that the *tat* mutant exhibited similar growth to WT strain (Figure 1b). Next, a mouse infection assay was performed to test whether the deletion of the Tat system had any effect on the virulence of the ExPEC strain. Mice were intraperitoneally injected with 6 × 10^6^ CFU of WT and Δ*tat* strains, respectively. It was shown that the survival rate of the mice infected with Δ*tat* strain kept 80% (4/5), while those infected with WT strain fell to 0 (0/5) within 5 days post-infection (Figure 1c). The ability of the WT and Δ*tat* cells to survive within mice was further tested. Mice were intraperitoneally injected with 6.7 × 10^5^ cells (a non-lethal dose) of WT and Δ*tat* strain, respectively. As shown in Figure 1c, the bacterial loads of the Δ*tat* strain in the brain, lung, spleen, and blood decreased drastically within 36 hours post-infection, and the cells were almost completely cleared at the end of the experiment. In contrast, a very high level of bacterial loads of WT strain were still observed in each organ at 36 hours post-infection (Figure 1d). The adhesion assay result further showed that the ability of the Δ*tat* strain to adhere to host cells was dramatically decreased compared with the WT strain (Figure 3a). In the whole blood bactericidal assay, it was shown that, instead of being killed, the WT strain could even grow in mouse blood. In contrast, the number of viable bacteria of the Δ*tat* strain significantly dropped after incubation in whole blood (Figure 3b). The ability of the Δ*tat* strain to survive in serum was also significantly lower than that of the WT strain (Figure 3c). These results strongly suggested that the Tat system was essential for the virulence of ExPEC.

(a). Cell morphology analysis. Cells of WT, Δ*tat* and its complement (CΔ*tat*) strains were grown to mid-log phase and stained with regular Gram staining procedures followed by imaging using a light microscope. (b). Growth curves. Cells of WT, Δ*tat* and CΔ*tat* strains were subcultured from overnight-grown cultures into LB and incubated at 37°C with shaking. Optical density at a wavelength of 600 nm was measured at each indicated time point. The assay was performed in triplicate. (c). Survival rate. Mice were intraperitoneally injected with 6 × 10^6^ CFU of WT and Δ*tat* strains, respectively. The survival rate was recorded for 5 days. (d). Bacterial load. Mice were intraperitoneally injected with 6.7 × 10^5^ cells (a non-lethal dose) of WT and Δ*tat* strain, respectively. The mice were euthanized at each indicated time points and the organs were collected, weighed, homogenized in sterile saline, and plated on to LB for cell counting. * represents *p* value <0.05; ** represents *p* value <0.01, *** represents *p* value <0.0001.

### Tat substrates prediction and mutants construction

It is the Tat-exported substrate proteins, instead of the Tat system itself, that function in bacterial pathogenesis. The Tat system recognizes its substrate proteins via the N-terminally located signal sequence containing an SRRxFLK motif [5,7]. So, we performed a bioinformatics prediction by using TatP 1.0 server (http://www.cbs.dtu.dk/services/TatP/)[41] to search for Tat substrate proteins encoded in the genome of ExPEC PCN033 strain. As listed in Table 2, a total of 25 Tat substrates were found. We next constructed a series of Tat-related mutants. To facilitate *in vivo* competitive infection assay, we used a chloramphenicol resistance cassette to replace the target genes that are not present in the middle of an operon. As NapA, DmsA, TorA, TorZ, FdnG, FdoG, and YedY have been reported to be functional only when cofactor molybdenum (Mo) is incorporated with and have an overlapping function in respiration, single deletion of each of these genes may not have an obvious phenotype [42–46]. Therefore, a Δ*moaA* mutant was constructed which was deficient in Mo cofactor biogenesis, in which the Tat substrates containing Mo as cofactor were functionally disrupted [36,47]. AmiA and AmiC are two amidases that are both exported through the Tat pathway and have overlapping functions [39]. Therefore, a double deletion mutant Δ*amiA*Δ*amiC* was also constructed.

**Table 2.** Predicted Tat substrates encoded in ExPEC PCN033 genome.

No.	Protein	Gene locus	Tat signal sequence	Predicted function
1	HyaA	PPECC33_RS05345	MNNEETFYQAMRRQGVTRRSFLKYCSLAA	Hydrogen oxidation
2	HybO	PPECC33_RS16535	MTGDNTLIHSHGINRRDFMKLCAALAATMGLSSKAAA	Hydrogen oxidation
3	HybA	PPECC33_RS16530	MNRRNFIKAASCGALLTGALPSVSHAA	Hydrogen oxidation
4	NapG	PPECC33_RS11870	MSRSAKPQNGRRRFLRDVVRTAGGLAAVGVALGLQQQTARA	Nitrate reduction
5	NrfC	PPECC33_RS22515	MTWSRRQFLTGVGVLAAVSGTAGRVVA	Nitrite reduction
6	YagT	PPECC33_RS01655	MSNQGEYPEDNRVGKHEPHDFSLTRRDLIKVSAATAATAVVYPHSTLAASVPA	Aldehyde oxidoreductase
7	YdhX	PPECC33_RS09020	MSFTRRKFVLGMGTVIFFTGSASSLLA	Unknown
8	TorA*	PPECC33_RS05440	MNNNDLFQASRRRFLAQLGGLTVAGMLGPSLLTPRRATAAQA	TMAO reduction
9	TorZ*	PPECC33_RS10195	MTLTRREFIKHSGIAAGALVVTSAAPLPAWA	TMAO reduction
10	NapA*	PPECC33_RS11875	MKLSRRSFMKANAVAAAAAAAGLSVPGVA	Nitrate reduction
11	DmsA*	PPECC33_RS04930	MKTKIPDAVLAAEVSRRGLVKTTAIGGLAMASSALTLPFSRIAHA	DMSO reduction
12	FdnG*	PPECC33_RS08015	MDVSRRQFFKICAGGMAGTTVAALGFAPKQALA	Formate oxidation
13	FdoG*	PPECC33_RS21300	MQVSRRQFFKICAGGMAGTTAAALGFAPSVALA	Formate oxidation
14	YedY*	PPECC33_RS10665	MKKNQFLKESDVTAESVFFMTRRQVLKALGISAAALSLPHAAHA	TMAO/DMSO reduction
15	CueO	PPECC33_RS00655	MQRRDFLKYSVALGVASALPLWSRAVFA	Copper homeostasis
16	SufI	PPECC33_RS16630	MSLSRRQFIQASGIALCAGAVPLKASA	Cell division
17	YahJ	PPECC33_RS01870	MKESNSRREFLSQSGKMVTAAALFGTSVPLAHA	Unknown
18	WcaM	PPECC33_RS11010	MPFKKLSRRTFLTASSALAFLHTPFARA	Colanic acid biosynthesis
19	MdoD	PPECC33_RS07765	MDRRRFIKGSMAMAAVCGTSGIASLFSQAAFA	Glucans biosynthesis
20	AmiA	PPECC33_RS12925	MSTFKPLKTLTSRRQVLKAGLAALTLSGMSQAIA	Cell wall remodeling
21	AmiC	PPECC33_RS15135	MSGSNTAISRRRLLQGAGAMWLLSVSQVSLA	Cell wall remodeling
22	FhuD	PPECC33_RS00805	MSGLPLISRRRLLTAMALSPLLWQMNTAQA	Ferrichrome binding
23	YcbK	PPECC33_RS05085	MDKFDANRRKLLALGGVALGAAILPTPAFA	Unknown
24	EfeO	PPECC33_RS05560	MTINFRRNALQLSVAALFSSAFMANA	Ferrous iron transport
25	EfeB	PPECC33_RS05565	MQYEDENGVNEPSRRRLLKGIGALALAGSCPVAHA	Ferrous iron transport

*Proteins that are reported to contain molybdenum as co-factor.

**Table 3.** Competitive index (n = 5).

Strain	Mean CI	*p* value	Significance
Δ*tat-Cm*	0.00568	6.01E-10	***
Δ*sufI*	0.11705	7.72E-05	***
Δ*amiA*Δ*amiC*	0.31352	0.00133	**
Δ*amiA*	0.38515	0.00281	**
Δ*amiC*	0.61580	0.00762	**
Δ*yahJ*	0.69739	0.02196	*
Δ*yagT*	0.81374	0.23846	NS
Δ*fhuD*	0.83035	0.19249	NS
Δ*cueO*	0.83868	0.02143	*
Δ*wcaM*	0.88963	0.46957	NS
Δ*efeOB*	0.90033	0.77743	NS
Δ*mdoD*	0.90440	0.62464	NS
Δ*fdoG*	0.92328	0.48688	NS
Δ*hybAO*	0.92962	0.36763	NS
Δ*napG*	0.93111	0.03731	*
Δ*fdnG*	0.98374	0.78275	NS
Δ*ydhX*	0.99369	0.86272	NS
Δ*nrfC*	1.06584	0.74763	NS
Δ*hyaA*	1.15042	0.27652	NS
Δ*moaA*	1.16533	0.22838	NS
Δ*ycbK*	1.56286	0.20617	NS

Note: n is the number of animals in each group. CI = Output (CFU_mutant_/CFU_WT_)/Input (CFU_mutant_/CFU_WT_). * indicates *p* value < 0.05; ** indicates *p* value < 0.01; *** indicates *p* value < 0.001. NS indicates no statistical significance.

### *Competitive infection assay to assess the* in vivo *fitness of Tat-related mutants*

To identify which Tat substrate protein accounted for the virulence attenuation of the *tat* mutant, we performed a series of competitive animal infection assays. As shown in Table 3, consistent with the result that disputing the Tat system significantly attenuated the virulence, the competitive infection assay also showed that the WT strain significantly outcompeted the Δ*tat* strain (CI = 0.00568, *p* < 0.0001). Seven out of the 20 mutants, including Δ*sufI*, Δ*amiA* Δ*amiC*, Δ*amiA*, Δ*amiC*, Δ*cueO*, Δ*yahJ*, and Δ*napG*, showed a CI less than 1 with statistical significance, indicating virulence attenuation compared with the WT strain. Among them Δ*sufI* displayed the lowest virulence, however, was not as avirulent as the Δ*tat* strain. Double deletion of *amiA* and *amiC* genes resulted in the second most attenuated mutant which showed a lower CI value than each single amidase mutant. Individual deletion of *yahJ*, encoding an uncharacterized protein, and *cueO*, encoding a blue copper oxidase, also led to somewhat virulence attenuation. The remaining mutants, including 10 Tat substrate mutants and the Δ*moaA* mutant, did not show significant virulence attenuation.

### SufI as well as AmiA and AmiC play an important role in pathogenesis of ExPEC

**Figure 2.**
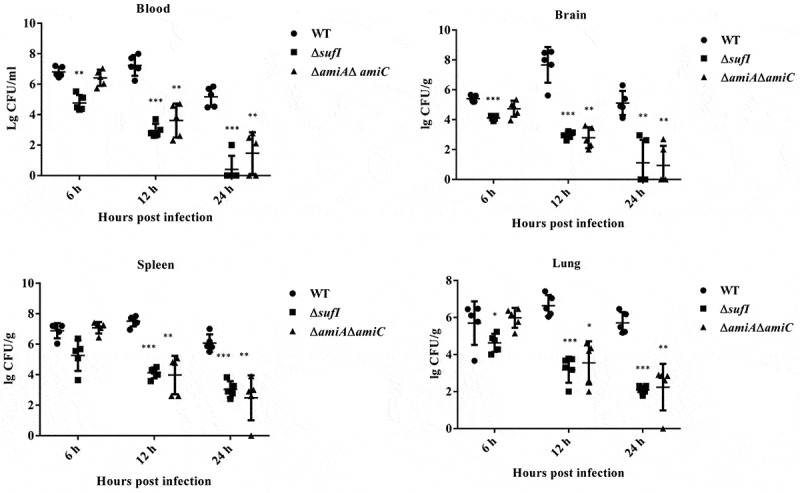
Colonization of WT, Δ*sufI*, and Δ*amiA*Δ*amiC* strains in mouse.

**Figure 3.**
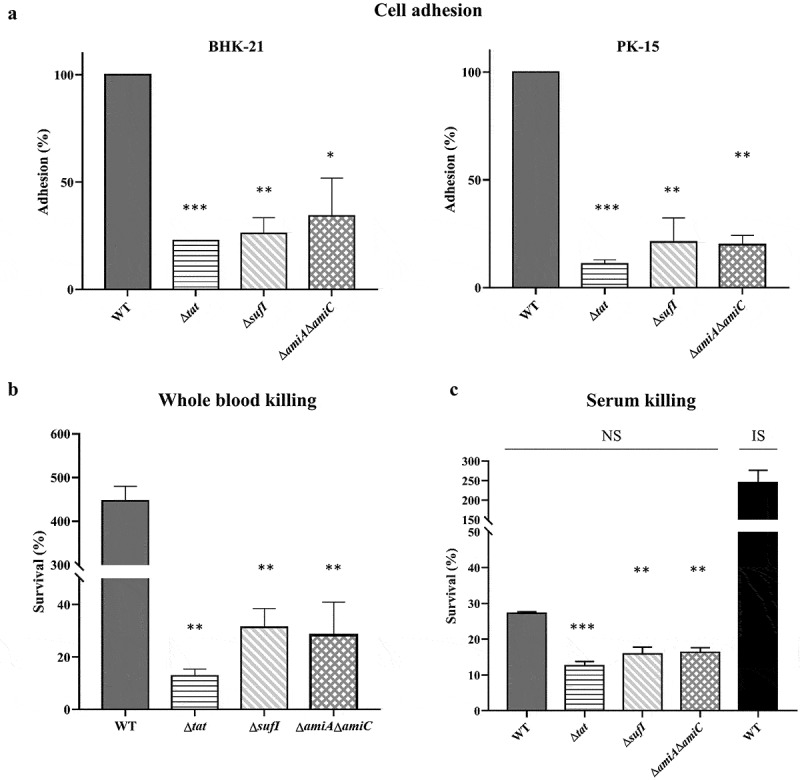
*In vitro* cell adhesion, and whole blood and serum bactericidal assays. (a). *In vitro* cell adhesion.

As Δ*sufI* and Δ*amiA* Δ*amiC* showed the lowest CI value in the competitive infection assay, individual infection experiment was carried out to further assess their virulence. Three groups of mice were intraperitoneally injected with 9.4 × 10^5^ CFU of WT, Δ*sufI*, Δ*amiA*Δ*amiC* strains, respectively. As shown in Figure 2, at 24 hours post-infection, a very high level of WT bacteria was present in each indicated organs. In contrast, both Δ*sufI* and Δ*amiA*Δ*amiC* strains were rapidly cleared *in vivo*. Growth assay showed that Δ*sufI* and Δ*amiA* Δ*amiC* strains exhibited a slightly slower growth rate than the WT strain during the log phase, but reached a similar cell density with the WT strain in the stationary phase (Figure 1b). We further compared the ability of cell adhesion, and the resistance to whole blood and serum killing between the WT and the *sufI* and Δ*amiA*Δ*amiC* strains. As shown in Figure 3a, the abilities of adhesion to PK-15 and BHK-21 cells of the Δ*sufI* and Δ*amiA*Δ*amiC* strains were significantly lower than that of the WT strain, but were comparable to that of the Δ*tat*. The whole blood bactericidal and serum bactericidal assays further revealed that the Δ*sufI* and Δ*amiA*Δ*amiC* strains were more vulnerable in blood and serum than the WT strain (Figure 3b,c). These results further confirmed that SufI, and the two amidases play an important role in the pathogenesis of ExPEC.

Mice were intraperitoneally injected with 9.4 × 10^5^ CFU of WT, Δ*sufI*, and Δ*amiA*Δ*amiC* strains, respectively. The mice were euthanized at each indicated time points and the organs were collected, weighed, homogenized in sterile saline, and plated on to LB for cell counting. * represents *p* value <0.05; ** represents *p* value <0.01, *** represents *p* value <0.0001.

PK-15 cells and BHK-21 cells grown in 6-well plates were infected with cells of each indicated bacterial strain grown to mid-log phase with a ratio of 10:1 followed by incubation at 37°C with 5% CO_2_ for 2 hours. The cells were then washed with PBS and lysed with sterile water. The input bacterial cells and the cell lysates were then diluted and plated onto LB plates for bacterial enumeration. The adhesion rate of the WT strain was set as 100%. (b). Whole blood bactericidal assay. Bacterial cells of each indicated strain grown to mid-log phase were incubated with heparinized mouse whole blood at 37°C for 1 hour. The initial input and the incubated samples were then diluted and plated onto LB plates for bacterial enumeration. (c). Serum blood bactericidal assay. Bacterial cells of each indicated strain grown to mid-log phase were incubated with normal mouse serum (NS) at 37°C for 1 hour. A control in which the WT strain was incubated with heat-inactivated serum (IS) was performed in parallel. The samples were then diluted and plated onto LB plates for bacterial enumeration. The initial input and the incubated samples were then diluted and plated onto LB plates for bacterial enumeration. The assays were performed in triplicate. The survival rate was calculated as (CFU_recovered_/CFU_input_) × 100. * represents *p* value <0.05; ** represents *p* value <0.01, *** represents *p* value <0.0001.

### *Motility was disrupted in the Δ*amiA*Δ*amiC *strain but not in the Δ*sufI

**Figure 4.**
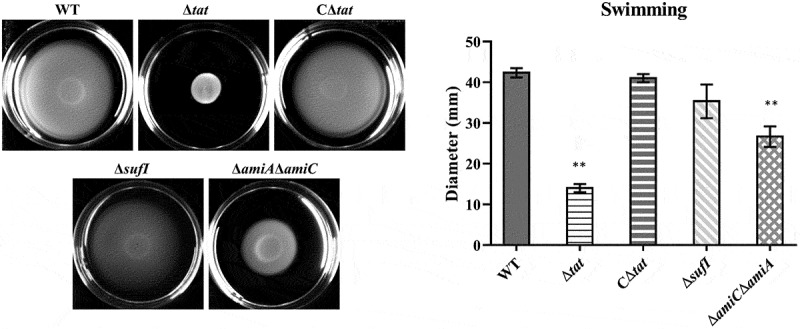
Motility assay.

By performing a bacterial swimming assay, we found that the deletion of the Tat system caused severe defects in the motility of ExPEC (Figure 4). As the motility-related functions have been recognized as a critical virulence factor [48–51], we next tested whether the motility phenotype of the Δ*sufI* and Δ*amiA* Δ*amiC* mutants was deficient as well, thus causing virulence attenuation. As shown in Figure 4, the swimming phenotype was significantly affected in the Δ*amiA* Δ*amiC* strain, although was not completely disrupted as that of the Δ*tat* strain. However, it was not disturbed in the Δ*sufI* strain. These results suggest that at least the virulence attenuation of the Δ*sufI* strain was not due to motility defect.

The cells of WT, Δ*tat*, CΔ*tat*, Δ*sufI*, CΔ*sufI* and Δ*amiA*Δ*amiC* strains were grown to mid-log phase and spotted onto agar plate containing 10 g/L tryptone, 5 g/L yeast extract, 5 g/L NaCl, 0.5% glucose (w/v), and 0.45% agar (w/v). The plate was photographed after incubation at 37°C for 8 h. The diameters of the zone were measured. The assay was performed in triplicate.

### *Δ*sufi *and Δ*amiA *Δ*amiC *displayed severe cell division defect in the log growth phase*

**Figure 5.**
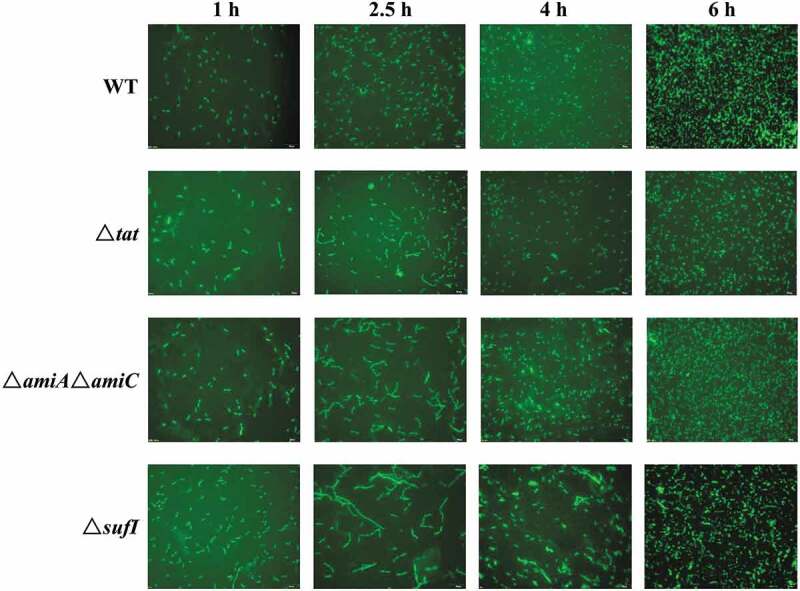
Live cell imaging of WT, Δ*tat*, CΔ*tat*, Δ*sufI*, and Δ*amiA*Δ*amiC* strains.

As SufI and the Tat-dependent amidases have been reported to be involved in cell division [52–54], the morphology of the mutant strains was analyzed. As shown in Figure 5, at 1 hour of growth, all the strains showed normal cell morphology. However, at the early log phase (2.5 hours of growth), in contrast to the wild-type strain that still displayed normal separated cells, all the Δ*tat*, Δ*sufI* and Δ*amiA* Δ*amiC* strains formed chained morphology suggesting abnormal cell division, among which the Δ*sufI* showed the most severe cell division defect. However, this chained morphology almost disappeared at 6 hours of growth. These results suggest that SufI, AmiA, and AmiC are critical for cell division, especially during the log phase.

Cells of WT, Δ*tat*, CΔ*tat*, Δ*sufI*, and Δ*amiA*Δ*amiC* strains containing pQE80Apra-GFP plasmid were cultured in LB at 37°C with shaking. Cells were taken at each indicated time point and imaged using a fluorescent microscope.

### *Δ*sufi *and Δ*amiA *Δ*amiC *were defective in stress response*

**Figure 6.**
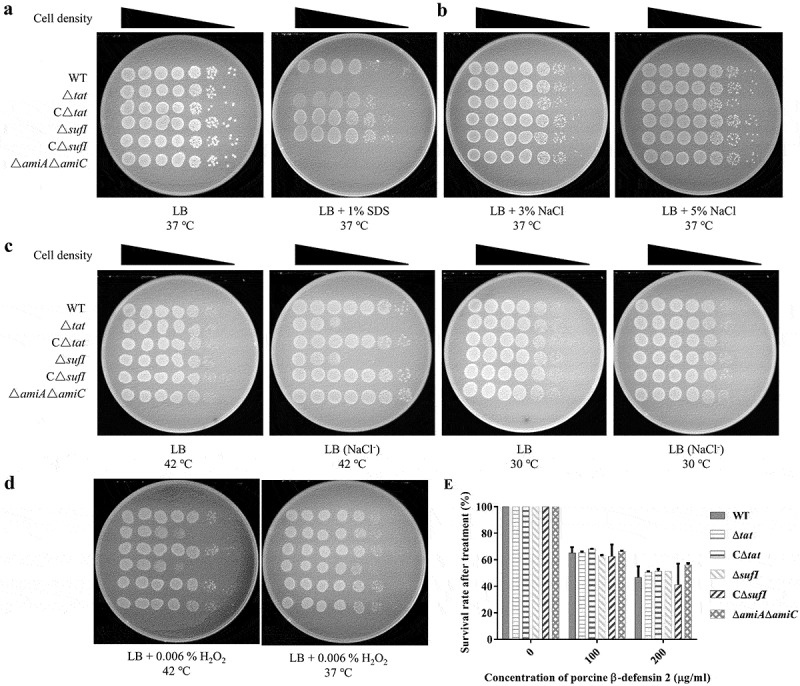
Stress response assay. (a). SDS resistance.

SDS sensitivity as an indicator of bacterial envelop integrity has been widely used, by which approach the Δ*tat* strain is shown to have a defective envelop [17,55,56]. Therefore, we tested whether the above mutants have a similar defect. As shown in Figure 6a, consistent with previous reports, both the Δ*tat* and Δ*amiA* Δ*amiC* showed high sensitivity to SDS, while the Δ*sufI* was as resistant as the WT strain. The growth of the strains in stress conditions was further investigated. Different stress conditions were generated, including high and low osmotic stresses, high-temperature stress, H_2_O_2_ induced oxidative stress, and porcine β-defensin 2 (PDB2) mediated antimicrobial stress. As shown in Figure 6b, all of Δ*tat*, Δ*sufI* and Δ*amiA* Δ*amiC* were able to grow in the medium containing up to 5% NaCl. However, Δ*sufI* exhibited impaired growth in LB medium without NaCl at 42°C but not at 30°C (Figure 6c). Furthermore, Δ*sufI* as well as Δ*tat* were sensitive to H_2_O_2_, while this sensitivity was only observed at 42°C, but not at 37°C (Figure 6d). None of Δ*tat*, Δ*sufI* and Δ*amiA* Δ*amiC* showed a different resistance to PDB2 (Figure 6e).

Cells of WT, Δ*tat*, CΔ*tat*, Δ*sufI*, CΔ*sufI*, and Δ*amiA*Δ*amiC* strains at mid-log phase were serially diluted and 3 μL of each culture was spotted onto LB plate containing 2% SDS which were incubated at 37°C overnight. (b). High osmotic stress response. Cells of WT, Δ*tat*, CΔ*tat*, Δ*sufI*, CΔ*sufI*, and Δ*amiA*Δ*amiC* strains at mid-log phase were serially diluted and 3 μL of each culture was spotted onto LB plate containing 3% or 5% NaCl which were incubated at 37°C overnight. (c). Low osmotic and high-temperature stresses response. Cells of WT, Δ*tat*, CΔ*tat*, Δ*sufI*, CΔ*sufI*, and Δ*amiA*Δ*amiC* strains at mid-log phase were serially diluted, and 3 μL of each culture was spotted onto LB plate with or without NaCl which were incubated at 30°C or 42°C overnight. (d). Oxidized stress response. Cells of WT, Δ*tat*, CΔ*tat*, Δ*sufI*, CΔ*sufI*, and Δ*amiA*Δ*amiC* strains at mid-log phase were serially diluted and mixed with 0.006% H_2_O_2_, and 3 μL of each culture was spotted onto LB plate with or without NaCl which were incubated at 30°C or 42°C overnight. E. Antimicrobial peptide resistance. 5 × 10^3^ CFU of cells of WT, Δ*tat*, CΔ*tat*, Δ*sufI*, CΔ*sufI*, and Δ*amiA*Δ*amiC* strains at mid-log phase were mixed with different concentrations of synthetic porcine β defensin at 37°C for 1 hour, and the samples were plated onto LB plates and the viable cells were counted. The assay was performed in triplicate.

## Discussion

In several important bacterial pathogens, the Tat system has been reported to be crucial for pathogenesis [8,14,17,35]. Consistently, our results also revealed that the deletion of the Tat system significantly attenuated the virulence of extra-intestinal pathogenic *E. coli* (ExPEC). In some pathogens, for example, *Legionella pneumophila* and *Pseudomonas aeruginosa*, the Tat system is involved in the secretion of exotoxins so as to contribute to virulence [57,58]. However, in the genome of the ExPEC PCN033 strain used in this study, we did not find any obvious virulence factors encompassing a Tat signal peptide through bioinformatics analysis.

In order to reveal how the Tat system affects bacterial pathogenesis, by constructing a total of 20 Tat-related mutants and performing competitive infection experiments, we demonstrated that several Tat substrate mutants exhibited significant virulence attenuation. Among these mutants, Δ*sufI* and Δ*amiA*Δ*amiC* showed the lowest CI value indicating the most severe virulence decrease. Similar findings have also been reported previously. In *Y. pseudotuberculosis*, several Tat substrates are identified critical to cause systematic infection, among which Δ*sufI* shows the largest attenuation [35]. In *Citrobacter freundii*, it has also been shown that *sufI* mutation results in a very significant *in vivo* fitness defect [15]. A recent study in *S*. Typhimurium demonstrates that the deletion of the two Tat-dependent amidase encoding genes leads to significant *in vivo* fitness attenuation, mainly due to cell division defect especially in the inflamed gut where stress conditions including high osmolarity and antimicrobial peptides are present [18]. However, a previous study which was also carried out in *S*. Typhimurium shows that neither the mutant devoid of *sufI* nor that lacking amidases alone, but the triple deletion mutant, display a defective virulence phenotype similar as the *tat* mutant, in which they propose that the virulence attenuation is primarily due to envelop defects [17].

Bacterial pathogens encounter a variety of stresses during infection, including antimicrobial peptides secreted by epithelial cells [59], hyperosmolarity [60], and ROS- or iNOS-mediated oxidative stress [61,62]. The ability of bacterial stress response contributes much to its *in* *vivo* fitness. In this study, Δ*amiA*Δ*amiC* and Δ*sufI* both showed significant virulence attenuation. SufI and the Tat-exported amidases are both involved in cell division of *E. coli. sufI* serves as a genetic suppressor of *ftsI* which encodes an essential cell division protein and has been shown to function under stress conditions [53,54,63]. AmiA and AmiC are N-acetylmuramyl-L-alanine amidases involved in cell wall remodeling [52]. Consistent with their critical roles in cell division, our data demonstrate that the two mutant strains exhibited abnormal morphology in the log growth phase. It is worth noting that although AmiA and AmiC are paralogs functioning in cell septation and separation, they contribute differently to virulence as the two single mutants gave different CI values in the competitive infection assay. This has also been observed in a previous study [17]. Actually, it has been reported that the subcellular localization of AmiA and AmiC differs markedly and they play different roles in cleaving the glycan chains of peptidoglycan [64,65]. Additionally, both of the Δ*amiA*Δ*amiC* and Δ*sufI* mutants are defective in stress response. Therefore, it is possible that the virulence attenuation in ExPEC caused by the disruption of the Tat substrates may be attributed to cell division defect-mediated compromise in stress response.

Bacterial motility has been recognized as an important virulence factor [48–51]. In consistence with the data reported in previous studies, our results also reveal that the disruption of the Tat system severely compromises bacterial motility. Therefore, we tested whether Δ*sufI* and Δ*amiA*Δ*amiC* possessed a similar non-motile phenotype as the Δ*tat* mutant, which may as a result account for the virulence attenuation. Our results showed that the motility was deficient in the Δ*amiA*Δ*amiC* strain, but not the Δ*sufI*. A similar motility defect in the Δ*amiA*Δ*amiC* strain has been also revealed in *S*. Typhimurium [17]. However, considering flagella expression is down-regulated during *S*. Typhimurium infection, the motility defect is not believed as the cause of virulence attenuation of the Δ*amiA*Δ*amiC* strain [17]. In ExPEC, our previous study has shown that the deletion of a flagella biosynthesis gene, *flgD*, causes significant virulence attenuation, indicating that motility is crucial for the virulence [51]. Therefore, whether and to which extent the motility defect can explain the virulence attenuation of the Δ*amiA*Δ*amiC* needs further investigations.

Δ*cueO* mutant was also significantly outcompeted by the WT strain in the competitive infection assay. *cueO* encodes a copper oxidase involved in bacterial copper homeostasis [66,67]. Although copper is an essential metal ion functioning as a cofactor of specific bacterial enzymes, too much copper is toxic. Copper is proposed to catalyze the production of hydroxyl radicals causing oxidative damage [68]. Excessive copper is also reported to interfere with disulfide bond formation in the periplasm of bacteria [69]. It has been shown that the copper level in the microenvironments within the host increases dramatically during bacterial infection [70]. Therefore, the ability of bacterial pathogens to tolerate high concentration copper may contribute to the pathogenicity. CueO itself is a copper-containing protein, therefore may contribute to copper efflux during its export through the Tat pathway which as a result decreases the copper concentration in the cytoplasm. Meanwhile, CueO, as an oxidase, is able to catalyze the toxic and membrane-permeable Cu(I) to the less toxic and less permeable Cu(II) [67,71]. Therefore, CueO may increase the copper tolerance of *E. coli* which contributes its survival *in vivo*.

Iron acquisition is another important physiological process linked to bacterial pathogenesis [10,72,73]. Bacteria have evolved different strategies to acquire iron, among which the Efe system is an iron transporter responsible for ferrous iron uptake [74]. The Efe system encompasses a membrane protein EfeU and two periplasmic proteins EfeO and EfeB which both bear a Tat signal peptide [74]. The Efe system, or its homologous systems, has been revealed to be required for the virulence of several important bacterial pathogens, including *Brucella abortus, Burkholderia* [75,76]. In the genome of *E. coli* K-12 MG1655 strain, there is a frameshift within the *efe* operon leading to the inactivation of this iron transporting function, and restoring this function by replacing the native *efeUOB* operon with an in-frame one increased the growth of *E. coli* under iron-restricted conditions [77]. However, in the genome of ExPEC PCN033, the *efeUOB* is intact without any frameshift, suggesting that it may be functional. Therefore, it is interesting to test whether the EfeUOB transporter is important for the pathogenesis of ExPEC. However, our results showed that Δ*efeOB* had a comparable virulence to the WT strain, indicating the EfeO and EfeB are not critical for the virulence and therefore not responsible for the virulence attenuation of the *tat* mutant.

A large proportion of the Tat substrates are co-factor containing redox enzymes, including trimethylamine N-oxide (TMAO) reductase TorA, dimethyl sulfoxide (DSMO) reductase DmsD, which mainly functions in electron transport chains in bacterial respiration [10]. In some pathogens, these redox enzymes have been shown to be crucial for virulence. In *Actinobacillus pleuropneumoniae*, the Δ*dmsA* mutant was significantly attenuated in a pig infection model [78]. In *Vibrio cholera*, deletion of *torD*, which encodes a chaperone protein required for the maturation of TorA, resulted in decrease in cholera toxin production and virulence, which was similar as the *tat* mutant [79]. In the genome of ExPEC PCN033 strain, seven Tat substrates, NapA, DmsA, TorA, TorZ, FdnG, FdoG, and YedY, are reported to be molybdenum-containing enzymes [45]. To assess the role of these enzymes in ExPEC virulence, instead of constructing every individual mutant, we deleted the *moaA* gene, which is responsible for molybdenum incorporation, therefore inactivates all of the above Tat substrates [44,47]. However, the animal infection experiment results showed that Δ*moaA* did not outcompete the WT strain, indicating no obvious virulence attenuation. Therefore, the molybdenum-containing Tat substrate proteins are not involved in the virulence attenuation.

In conclusion, our results demonstrate that the Tat system is critical for virulence of ExPEC, and SufI as well as AmiA and AmiC, are the key Tat substrates responsible for the virulence attenuation.

## Supplementary Material

Supplemental Material

Supplemental Material

## References

[1] Tseng TT, Tyler BM, Setubal JC. Protein secretion systems in bacterial-host associations, and their description in the gene ontology. BMC Microbiol. 2009;9(Suppl 1):S2. 19278550 10.1186/1471-2180-9-S1-S2 PMC2654662

[2] Green ER, Mecsas J. Bacterial secretion systems: an overview. Microbiol Spectr. 2016;4. 10.1128/microbiolspec.VMBF-0012-2015 PMC4804464 26999395

[3] Bhoite S, van Gerven N, Chapman MR, et al. Curli biogenesis: bacterial amyloid assembly by the type VIII secretion pathway. EcoSal Plus. 2019;8. 10.1128/ecosalplus.esp-0037-2018 PMC6428212 30892177

[4] Lauber F, Deme JC, Lea SM, et al. Type 9 secretion system structures reveal a new protein transport mechanism. Nature. 2018;564:77. 30405243 10.1038/s41586-018-0693-y PMC6927815

[5] Palmer T, Berks BC. The twin-arginine translocation (Tat) protein export pathway. Nature Rev Microbiol. 2012;10:483–496. 22683878 10.1038/nrmicro2814

[6] Berks BC. The twin-arginine protein translocation pathway. Annu Rev Biochem. 2015;84:843–864. 25494301 10.1146/annurev-biochem-060614-034251

[7] Berks BC. A common export pathway for proteins binding complex redox cofactors? Mol Microbiol. 1996;22:393–404. 8939424 10.1046/j.1365-2958.1996.00114.x

[8] Tullman-Ercek D, DeLisa MP, Kawarasaki Y, et al. Export pathway selectivity of Escherichia coli twin arginine translocation signal peptides. J Biol Chem. 2007;282:8309–8316. 17218314 10.1074/jbc.M610507200 PMC2730154

[9] Berks BC, Palmer T, Sargent F. The Tat protein translocation pathway and its role in microbial physiology. Adv Microb Physiol. 2003;47:187–254. 14560665 10.1016/s0065-2911(03)47004-5

[10] Palmer T, Sargent F, Berks BC. The Tat protein export pathway. EcoSal Plus. 2010;4. 10.1128/ecosalplus.4.3.2 26443788

[11] Ochsner UA, Snyder A, Vasil AI, et al. Effects of the twin-arginine translocase on secretion of virulence factors, stress response, and pathogenesis. Proc Natl Acad Sci U S A. 2002;99:8312–8317. 12034867 10.1073/pnas.082238299 PMC123064

[12] Pradel N, Ye C, Livrelli V, et al. Contribution of the twin arginine translocation system to the virulence of enterohemorrhagic Escherichia coli O157:H7. Infect Immun. 2003;71:4908–4916. 12933832 10.1128/IAI.71.9.4908-4916.2003 PMC187321

[13] Reynolds MM, Bogomolnaya L, Guo J, et al. Abrogation of the twin arginine transport system in Salmonella enterica serovar Typhimurium leads to colonization defects during infection. PloS One. 2011;6:e15800. 21298091 10.1371/journal.pone.0015800 PMC3027627

[14] Urrutia IM, Sabag A, Valenzuela C, et al. Contribution of the twin-arginine translocation system to the intracellular survival of salmonella typhimurium in dictyostelium discoideum. Front Microbiol. 2018;9:3001. 30574134 10.3389/fmicb.2018.03001 PMC6291500

[15] Anderson MT, Mitchell LA, Zhao L, et al. Citrobacter freundii fitness during bloodstream infection. Sci Rep. 2018;8:11792. 30087402 10.1038/s41598-018-30196-0 PMC6081441

[16] Lavander M, Ericsson SK, Broms JE, et al. The twin arginine translocation system is essential for virulence of Yersinia pseudotuberculosis. Infect Immun. 2006;74:1768–1776. 16495550 10.1128/IAI.74.3.1768-1776.2006 PMC1418654

[17] Craig M, Sadik AY, Golubeva YA, et al. Twin-arginine translocation system (tat) mutants of Salmonella are attenuated due to envelope defects, not respiratory defects. Mol Microbiol. 2013;89:887–902. 23822642 10.1111/mmi.12318 PMC3811912

[18] Fujimoto M, Goto R, Hirota R, et al. Tat-exported peptidoglycan amidase-dependent cell division contributes to Salmonella Typhimurium fitness in the inflamed gut. PLoS Pathog. 2018;14:e1007391. 30379938 10.1371/journal.ppat.1007391 PMC6231687

[19] Bronstein PA, Marrichi M, Cartinhour S, et al. Identification of a twin-arginine translocation system in Pseudomonas syringae pv. tomato DC3000 and its contribution to pathogenicity and fitness. J Bacteriol. 2005;187:8450–8461. 16321949 10.1128/JB.187.24.8450-8461.2005 PMC1317023

[20] Biran D, Ron EZ. Extraintestinal pathogenic Escherichia coli. Curr Top Microbiol Immunol. 2018;416:149–161. 30046982 10.1007/82_2018_108

[21] Liu CM, Stegger M, Aziz M, et al. Escherichia coli ST131-H22 as a foodborne uropathogen. mBio. 2018;9. 10.1128/mBio.00470-18 PMC6113624 30154256

[22] Bergeron CR, Prussing C, Boerlin P, et al. Chicken as reservoir for extraintestinal pathogenic Escherichia coli in humans, Canada. Emerg Infect Dis. 2012;18:415–421. 22377351 10.3201/eid1803.111099 PMC3309577

[23] Maluta RP, Logue CM, Casas MR, et al. Overlapped sequence types (STs) and serogroups of avian pathogenic (APEC) and human extra-intestinal pathogenic (ExPEC) Escherichia coli isolated in Brazil. PloS One. 2014;9:e105016. 25115913 10.1371/journal.pone.0105016 PMC4130637

[24] Nandanwar N, Janssen T, Kuhl M, et al. Extraintestinal pathogenic Escherichia coli (ExPEC) of human and avian origin belonging to sequence type complex 95 (STC95) portray indistinguishable virulence features. Int J Med Microbiol IJMM. 2014;304:835–842. 25037925 10.1016/j.ijmm.2014.06.009

[25] Ge X Z, Jiang J, Pan Z, et al. Comparative genomic analysis shows that avian pathogenic Escherichia coli isolate IMT5155 (O2: K1:H5;ST complex 95, ST140) shares close relationship with ST95 APEC O1: k1and human ExPEC O18: k1strains. PloS One. 2014;9:e112048. 25397580 10.1371/journal.pone.0112048 PMC4232414

[26] Gao J, Duan X, Li X, et al. Emerging of a highly pathogenic and multi-drug resistant strain of Escherichia coli causing an outbreak of colibacillosis in chickens. Infect Genet Evol. 2018;65:392–398. 30157463 10.1016/j.meegid.2018.08.026

[27] Tan C, Xu Z, Zheng H, et al. Genome sequence of a porcine extraintestinal pathogenic Escherichia coli strain. J Bacteriol. 2011;193:5038. 21742868 10.1128/JB.05551-11 PMC3165708

[28] Liu C, Zheng H, Yang M, et al. Genome analysis and in vivo virulence of porcine extraintestinal pathogenic Escherichia coli strain PCN033. BMC Genomics. 2015;16:717. 26391348 10.1186/s12864-015-1890-9 PMC4578781

[29] Tan C, Tang X, Zhang X, et al. Serotypes and virulence genes of extraintestinal pathogenic Escherichia coli isolates from diseased pigs in China. Vet J. 2012;192:483–488. 22036869 10.1016/j.tvjl.2011.06.038

[30] Zong B, Liu W, Zhang Y, et al. Effect of kpsM on the virulence of porcine extraintestinal pathogenic Escherichia coli. FEMS Microbiol Lett. 2016;363. 10.1093/femsle/fnw232 27737948

[31] Roland K, Curtiss R 3rd, Sizemore D. Construction and evaluation of a delta cya delta crp Salmonella typhimurium strain expressing avian pathogenic Escherichia coli O78 LPS as a vaccine to prevent airsacculitis in chickens. Avian Dis. 1999;43:429–441. 10494411

[32] Edwards RA, Keller LH, Schifferli DM. Improved allelic exchange vectors and their use to analyze 987P fimbria gene expression. Gene. 1998;207:149–157.9511756

[33] Platt R, Drescher C, Park SK, et al. Genetic system for reversible integration of DNA constructs and lacZ gene fusions into the Escherichia coli chromosome. Plasmid. 2000;43:12–23. 10610816 10.1006/plas.1999.1433

[34] Macho AP, Rufian JS, Ruiz-Albert J, et al. Competitive index: mixed infection-based virulence assays for genetic analysis in pseudomonas syringae-plant interactions. Methods Mol Biol. 2016;1363:209–217. 26577792 10.1007/978-1-4939-3115-6_17

[35] Avican U, Doruk T, Ostberg Y, et al. The Tat substrate sufi is critical for the ability of yersinia pseudotuberculosis to cause systemic infection. Infect Immun. 2017;85. 10.1128/IAI.00867-16 PMC5364315 28115509

[36] Hughes ER, Winter MG, Duerkop BA, et al. Microbial respiration and formate oxidation as metabolic signatures of inflammation-associated dysbiosis. Cell Host Microbe. 2017;21:208–219. 28182951 10.1016/j.chom.2017.01.005 PMC5313043

[37] Zong B, Zhang Y, Wang X, et al. Characterization of multiple type-VI secretion system (T6SS) VgrG proteins in the pathogenicity and antibacterial activity of porcine extra-intestinal pathogenic Escherichia coli. Virulence. 2019;10:118–132. 30676217 10.1080/21505594.2019.1573491 PMC6363058

[38] Wang H, Liu L, Cao Q, et al. Haemophilus parasuis alpha-2,3-sialyltransferase-mediated lipooligosaccharide sialylation contributes to bacterial pathogenicity. Virulence. 2018;9:1247–1262. 30036124 10.1080/21505594.2018.1502606 PMC6104685

[39] Ize B, Stanley NR, Buchanan G, et al. Role of the Escherichia coli Tat pathway in outer membrane integrity. Mol Microbiol. 2003;48:1183–1193. 12787348 10.1046/j.1365-2958.2003.03504.x

[40] Alcock F, Stansfeld PJ, Basit H, et al. Assembling the Tat protein translocase. Elife. 2016;5. 10.7554/eLife.20718 PMC5201420 27914200

[41] Bendtsen JD, Nielsen H, Widdick D, et al. Prediction of twin-arginine signal peptides. BMC Bioinformatics. 2005;6:167. 15992409 10.1186/1471-2105-6-167 PMC1182353

[42] Cheng VW, Weiner JH. S- and N-oxide reductases. EcoSal Plus. 2007;2. 10.1128/ecosalplus.3.2.8 26443585

[43] Cole JA, Richardson DJ. Respiration of nitrate and nitrite. EcoSal Plus. 2008;3. 10.1128/ecosal.3.2.5 26443731

[44] Magalon A, Mendel RR. Biosynthesis and insertion of the molybdenum cofactor. EcoSal Plus. 2008;3. 10.1128/ecosalplus.3.6.3.13 26443739

[45] Leimkuhler S, Iobbi-Nivol C. Bacterial molybdoenzymes: old enzymes for new purposes. FEMS Microbiol Rev. 2016;40:1–18. 26468212 10.1093/femsre/fuv043

[46] Pinske C, Sawers RG. Anaerobic formate and hydrogen metabolism. EcoSal Plus. 2016;7. 10.1128/ecosalplus.esp-0011-2016 PMC11575713 27735784

[47] Rivers SL, McNairn E, Blasco F, et al. Molecular genetic analysis of the moa operon of Escherichia coli K-12 required for molybdenum cofactor biosynthesis. Mol Microbiol. 1993;8:1071–1081. 8361352 10.1111/j.1365-2958.1993.tb01652.x

[48] Chaban B, Hughes HV, Beeby M. The flagellum in bacterial pathogens: for motility and a whole lot more. Semin Cell Dev Biol. 2015;46:91–103. 26541483 10.1016/j.semcdb.2015.10.032

[49] Erhardt M. Strategies to block bacterial pathogenesis by interference with motility and chemotaxis. Curr Top Microbiol Immunol. 2016;398:185–205. 27000091 10.1007/82_2016_493

[50] Weller-Stuart T, Toth I, De Maayer P, et al. Swimming and twitching motility are essential for attachment and virulence of Pantoea ananatis in onion seedlings. Mol Plant Pathol. 2017;18:734–745. 27226224 10.1111/mpp.12432 PMC6638301

[51] Liu F, Fu J, Liu C, et al. Characterization and distinction of two flagellar systems in extraintestinal pathogenic Escherichia coli PCN033. Microbiol Res. 2017;196:69–79. 28164791 10.1016/j.micres.2016.11.013

[52] Heidrich C, Templin MF, Ursinus A, et al. Involvement of N-acetylmuramyl-L-alanine amidases in cell separation and antibiotic-induced autolysis of Escherichia coli. Mol Microbiol. 2001;41:167–178. 11454209 10.1046/j.1365-2958.2001.02499.x

[53] Samaluru H, SaiSree L, Reddy M. Role of SufI (FtsP) in cell division of Escherichia coli: evidence for its involvement in stabilizing the assembly of the divisome. J Bacteriol. 2007;189:8044–8052. 17766410 10.1128/JB.00773-07 PMC2168700

[54] Tarry M, Arends SJ, Roversi P, et al. The Escherichia coli cell division protein and model Tat substrate SufI (FtsP) localizes to the septal ring and has a multicopper oxidase-like structure. J Mol Biol. 2009;386:504–519. 19135451 10.1016/j.jmb.2008.12.043 PMC2661564

[55] Huang Q, Alcock F, Kneuper H, et al. A signal sequence suppressor mutant that stabilizes an assembled state of the twin arginine translocase. Proc Natl Acad Sci U S A. 2017;114:E1958–E67. 28223511 10.1073/pnas.1615056114 PMC5347605

[56] Huang Q, Palmer T. Signal peptide hydrophobicity modulates interaction with the twin-arginine translocase. mBio. 2017;8. 10.1128/mBio.00909-17 PMC5539426 28765221

[57] Voulhoux R, Ball G, Ize B, et al. Involvement of the twin-arginine translocation system in protein secretion via the type II pathway. Embo J. 2001;20:6735–6741. 11726509 10.1093/emboj/20.23.6735 PMC125745

[58] Rossier O, Cianciotto NP. The Legionella pneumophila tatB gene facilitates secretion of phospholipase C, growth under iron-limiting conditions, and intracellular infection. Infect Immun. 2005;73:2020–2032. 15784543 10.1128/IAI.73.4.2020-2032.2005 PMC1087389

[59] Xia X, Cheng L, Zhang S, et al. The role of natural antimicrobial peptides during infection and chronic inflammation. Antonie Van Leeuwenhoek. 2018;111:5–26. 28856473 10.1007/s10482-017-0929-0

[60] Henderson AG, Ehre C, Button B, et al. Cystic fibrosis airway secretions exhibit mucin hyperconcentration and increased osmotic pressure. J Clin Invest. 2014;124:3047–3060. 24892808 10.1172/JCI73469 PMC4072023

[61] Dryden M. Reactive oxygen species: a novel antimicrobial. Int J Antimicrob Agents. 2018;51:299–303. 28887201 10.1016/j.ijantimicag.2017.08.029

[62] Fang FC, Vazquez-Torres A. Reactive nitrogen species in host-bacterial interactions. Curr Opin Immunol. 2019;60:96–102. 31200187 10.1016/j.coi.2019.05.008 PMC6800629

[63] Spratt BG. Temperature-sensitive cell division mutants of Escherichia coli with thermolabile penicillin-binding proteins. J Bacteriol. 1977;131:293–305. 326764 10.1128/jb.131.1.293-305.1977 PMC235422

[64] Bernhardt TG, de Boer PA. The Escherichia coli amidase AmiC is a periplasmic septal ring component exported via the twin-arginine transport pathway. Mol Microbiol. 2003;48:1171–1182. 12787347 10.1046/j.1365-2958.2003.03511.x PMC4428285

[65] Priyadarshini R, Popham DL, Young KD. Daughter cell separation by penicillin-binding proteins and peptidoglycan amidases in Escherichia coli. J Bacteriol. 2006;188:5345–5355. 16855223 10.1128/JB.00476-06 PMC1540038

[66] Grass G, Rensing C. CueO is a multi-copper oxidase that confers copper tolerance in Escherichia coli. Biochem Biophys Res Commun. 2001;286:902–908. 11527384 10.1006/bbrc.2001.5474

[67] Djoko KY, Chong LX, Wedd AG, et al. Reaction mechanisms of the multicopper oxidase CueO from Escherichia coli support its functional role as a cuprous oxidase. J Am Chem Soc. 2010;132:2005–2015. 20088522 10.1021/ja9091903

[68] Liochev SI, Fridovich I. The Haber-Weiss cycle – 70 years later: an alternative view. Redox Rep. 2002;7:55–57. 11981456 10.1179/135100002125000190

[69] Hiniker A, Collet JF, Bardwell JC. Copper stress causes an in vivo requirement for the Escherichia coli disulfide isomerase DsbC. J Biol Chem. 2005;280:33785–33791. 16087673 10.1074/jbc.M505742200

[70] Wagner D, Maser J, Lai B, et al. Elemental analysis of Mycobacterium avium-, Mycobacterium tuberculosis-, and Mycobacterium smegmatis-containing phagosomes indicates pathogen-induced microenvironments within the host cell’s endosomal system. J Iimmunol. 2005;174:1491–1500. 10.4049/jimmunol.174.3.1491 15661908

[71] Outten FW, Huffman DL, Hale JA, et al. The independent cue and cus systems confer copper tolerance during aerobic and anaerobic growth in Escherichia coli. J Biol Chem. 2001;276:30670–30677. 11399769 10.1074/jbc.M104122200

[72] Sheldon JR, Laakso HA, Heinrichs DE. Iron acquisition strategies of bacterial pathogens. Microbiol Spectr. 2016;4. 10.1128/microbiolspec.VMBF-0010-2015 27227297

[73] Lemos ML, Balado M. Iron uptake mechanisms as key virulence factors in bacterial fish pathogens. J Appl Microbiol. 2020;129:104–115. 31994331 10.1111/jam.14595

[74] Grosse C, Scherer J, Koch D, et al. A new ferrous iron-uptake transporter, EfeU (YcdN), from Escherichia coli. Mol Microbiol. 2006;62:120–131. 16987175 10.1111/j.1365-2958.2006.05326.x

[75] Elhassanny AE, Anderson ES, Menscher EA, et al. The ferrous iron transporter FtrABCD is required for the virulence of Brucella abortus 2308 in mice. Mol Microbiol. 2013;88:1070–1082. 23647104 10.1111/mmi.12242

[76] Mathew A, Eberl L, Carlier AL. A novel siderophore-independent strategy of iron uptake in the genus Burkholderia. Mol Microbiol. 2014;91:805–820. 24354890 10.1111/mmi.12499

[77] Cao J, Woodhall MR, Alvarez J, et al. EfeUOB (YcdNOB) is a tripartite, acid-induced and CpxAR-regulated, low-pH Fe2+ transporter that is cryptic in Escherichia coli K-12 but functional in E. coli O157: H7. Mol Microbiol. 2007;65:857–875. 17627767 10.1111/j.1365-2958.2007.05802.x

[78] Baltes N, Hennig-Pauka I, Jacobsen I, et al. Identification of dimethyl sulfoxide reductase in Actinobacillus pleuropneumoniae and its role in infection. Infect Immun. 2003;71:6784–6792. 14638764 10.1128/IAI.71.12.6784-6792.2003 PMC308893

[79] Lee KM, Park Y, Bari W, et al. Activation of cholera toxin production by anaerobic respiration of trimethylamine N-oxide in Vibrio cholerae. J Biol Chem. 2012;287:39742–39752. 23019319 10.1074/jbc.M112.394932 PMC3501055

