# Supplementary material for: The Tat system and its dependent cell division proteins are critical for virulence of extra-intestinal pathogenic Escherichia coli
Source: Virulence. 2020 Sep 22;11(1):1279–92. doi: 10.1080/21505594.2020.1817709 (PMC7549933; doi:10.1080/21505594.2020.1817709)
Supplement: Supplemental Material [file KVIR_A_1817709_SM9473.docx]

**Table S2. Plasmid information**

| **Plasmid** | **Description** | **Source** |
| --- | --- | --- |
| pRE112 | Suicide vector, *oriT*, *oriV*, *sacB*, Cm^R^ | Lab stock |
| pHSG396 | Cloning vector, Cm^R^ | Takara Bio Inc. |
| pQE80L | Expression vector | Lab stock |
| pRE112-Δ*tatABC*1 | Complement vector of *tatABC* gene | This study |
| pRE112-Δ*tatABC*2 | pRE112 containing 1000 bp sequence upstream *tatA* followed by 1000 bp downstream *tatC.* | This study |
| pRE112-Δ*amiA* | pRE112 containing a chloramphenicol resistance cassette flanked by 1000 bp upstream and downstream of *amiA* | This study |
| pRE112-Δ*amiA*2 | pRE112 containing 1000 bp sequence upstream *amiA* followed by 1000 bp downstream *amiA.* | This study |
| pRE112-Δ*amiC* | pRE112 containing a chloramphenicol resistance cassette flanked by 1000 bp upstream and downstream of *amiC* | This study |
| pRE112-Δ*sufI* | pRE112 containing a chloramphenicol resistance cassette flanked by 1000 bp upstream and downstream of *sufI* | This study |
| pRE112-Δ*moaA* | pRE112 containing chloramphenicol resistance cassette flanked by 1000 bp upstream and downstream of *moaA* | This study |
| pRE112-Δ*cueO* | pRE112 containing a chloramphenicol resistance cassette flanked by 1000 bp upstream and downstream of *cueO* | This study |
| pRE112-Δ*yahJ* | pRE112 containing a chloramphenicol resistance cassette flanked by 1000 bp upstream and downstream of *yahJ* | This study |
| pRE112-Δ*wcaM* | pRE112 containing a chloramphenicol resistance cassette flanked by 1000 bp upstream and downstream of *wcaM* | This study |
| pRE112-Δ*modD* | pRE112 containing a chloramphenicol resistance cassette flanked by 1000 bp upstream and downstream of *mdoD* | This study |
| pRE112-Δ*fhuD* | pRE112 containing a chloramphenicol resistance cassette flanked by 1000 bp upstream and downstream of *fhuD* | This study |
| pRE112-Δ*ycbK* | pRE112 containing a chloramphenicol resistance cassette flanked by 1000 bp upstream and downstream of *ycbK* | This study |
| pRE112-Δ*efeOB* | pRE112 containing a chloramphenicol resistance cassette flanked by 1000 bp upstream and downstream of *efeOB* | This study |
| pRE112-Δ*fdnG* | pRE112 containing a chloramphenicol resistance cassette flanked by 1000 bp upstream and downstream of *fdnG* | This study |
| pRE112-Δ*fdoG* | pRE112 containing a chloramphenicol resistance cassette flanked by 1000 bp upstream and downstream of *fdoG* | This study |
| pRE112-Δ*hyaA* | pRE112 containing a chloramphenicol resistance gene coding sequence flanked by 1000 bp upstream and downstream of *hyaA* coding sequence. | This study |
| pRE112-Δ*napG* | pRE112 containing a chloramphenicol resistance cassette flanked by 1000 bp upstream and downstream of *napG.* | This study |
| pRE112-Δ*hybAO* | pRE112 containing a chloramphenicol resistance cassette flanked by 1000 bp upstream *hybA* and downstream *hybO.* | This study |
| pRE112-Δ*nrfC* | pRE112 containing a chloramphenicol resistance gene coding sequence flanked by 1000 bp upstream and downstream of *nrfC* coding sequence. | This study |
| pRE112-Δ*yagT* | pRE112 containing a chloramphenicol resistance gene coding sequence flanked by 1000 bp upstream and downstream of *yagT* coding sequence. | This study |
| pRE112-Δ*ydhX* | pRE112 containing a chloramphenicol resistance gene coding sequence flanked by 1000 bp upstream and downstream of *ydhX* coding sequence. | This study |
| pHSG396Apra | As pHSG396, the chloramphenicol resistance cassette replaced with an apramycin resistance cassette. | This study |
| pHSG-*tatABC* | pHSG396 containing *tatABC* with its promoter region. |  |
| pHSG396Apra-*sufI* | pHSG396Apra cloned with tat promoter followed by *sufI* coding sequence. | This study |
| pQE80Apra | As pQE80L, the ampcillin resistance cassette replaced with an apramycin resistance cassette. | This study |
| pQE80Apra-GFP | pQE80Apra cloned with *egfp* coding sequence. | This study |
