# Supplementary material for: The Tat system and its dependent cell division proteins are critical for virulence of extra-intestinal pathogenic Escherichia coli
Source: Virulence. 2020 Sep 22;11(1):1279–92. doi: 10.1080/21505594.2020.1817709 (PMC7549933; doi:10.1080/21505594.2020.1817709)
Supplement: Supplemental Material [file KVIR_A_1817709_SM9472.docx]

**Table S1. Primer information**

| **Primer name** | **Sequence (5’ to 3’)** |
| --- | --- |
| tatA1 up1 | ACTGCATGAATTCCCGGGAGAGCTCGATGGTGATGGAGCGCATT |
| tatA1 up2 | GCCCTGACGGGCGGTTGAAT ACATGTTCCTCTGTGGTAGAT |
| tatC1 down1 | CTACCACAGA GGAACATGT ATT CAACCGCCCG TCAGGG |
| tatC1 down2 | AGGTACCGCATGCGATATCGAGCTCATTGTCGATCCGGCAACCC |
| tatA up150-F | GGCTGATGCCCGGCTGGTTA |
| tatC down150-R | TAGTGCCGGTGATGAGTAGC |
| pHSGTatABC-F | CTCGGTACCCCATCGATGGGGGATCCTGTCGGTTGGCGCAAAACA |
| pHSGTatABC-R | GCTTGCATGCCCCTCGAGGGGTCGACTTATTCTTCAGTTTTTTCGCTTT |
| M13-F | GTAAAACGACGGCCAGT |
| M13-R | GGAAACAGCTATGACCATG |
| PRE112-F | TGTAAGTGAACTGCATGAATT |
| pRE112-R | TGCTACGCCTGAATAAGT |
| TatA2 up1 | TAAGTGAACTGCATGAATTCCCGGGGATGGTGATGG AGCGCATTT |
| TatA2 up2 | AACCTCTTACGTGCCGATCAACATGTTCCTCTGTGGTAGAT |
| TatA2-Chl-1 | CTACCACAGA GGAACATGTTGATCGGCACGTAAGAGGTT |
| TatC2-Chl-2 | GCCCTGACGGGCGGTTGAATTTACGCCCCGCCCTGCCA |
| TatC2down1 | AGTGGCAGGGCGGGGCGTAAATTCAACCGCCCG TCAGG |
| TatC2down2 | GGCCCGATCCCAAGCTTCTTCTAGAATTGTCGATCCGGCAACCC |
| amiA up1 | TAAGTGAACTGCATGAATTCCCGGGCACTACCAGCGACAGACCGA |
| amiA up2 | AACCTCTTACGTGCCGATCA ACGGCCTGAGTTGTAATAAT AAA |
| amiA -chl-1 | ATTATTACAACTCAGGCCGTTGATCGGCACGTAAGAGGTT |
| amiA -chl-2 | TGTGCGTCGGGTTTCATCACTTACGCCCCGCCCTGCCA |
| amiA down1 | AGTGGCAGGGCGGGGCGTAAGTGATGAAACCCGACGCACA |
| amiA down2 | GGCCCGATCCCAAGCTTCTTCTAGAGGCATCTGGGGCAATTTGC |
| amiA-out-F | TATCTCACGAAAATCGGTT |
| amiA-out-R | CAACACCCGGCTACGC |
| amiA-in-F | ATGAGCACTTTTAAACCACT |
| amiA-in-R | TTACCGCTTTCTCGAGTGT |
| sufI up1 | TAAGTGAACTGCATGAATTCCCGGGGATGAAACGTTAAGTCGTCAT |
| sufI up2 | AACCTCTTACGTGCCGATCA ATTTGCTCCATGTAAAACTGGCT |
| sufI-chl-1 | CAGTTTTACATGGAGCAAATTGATCGGCACGTAAGAGGTT |
| sufI-chl-2 | TCCTCGGGCGAGTATGAAGATTACGCCCCGCCCTGCCA |
| sufI down1 | AGTGGCAGGGCGGGGCGTAATCTTCATACTCGCCCGAGGA |
| sufI down2 | GGCCCGATCCCAAGCTTCTTCTAGAAAAGAAGGCAGCAATACGTA |
| sufI-out-F | TCGTTCGATAATGGAACAAAA |
| sufI-out-R | ACAGGTCGCGATCCGGTT |
| sufI-in-F | ATGTCACTCAGTCGGCGT |
| sufI-in-R | TTACGGTACCGGGTTGACT |
| amiC up1 | TAAGTGAACTGCATGAATTCCCGGGCTTTCACTGCGCCACGCAAA |
| amiC up2 | AACCTCTTACGTGCCGATCAGCCTCTCCCGAGTAAAAGAA |
| amiC-chl-1 | TTCTTTTACTCGGGAGAGGCTGATCGGCACGTAAGAGGTT |
| amiC-chl-2 | CGCCATTCAGCGCCTTTTTATTACGCCCCGCCCTGCCA |
| amiCdown1 | AGTGGCAGGGCGGGGCGTAATAAAAAGGCGCTGAATGGCG |
| amiCdown2 | GGCCCGATCCCAAGCTTCTTCTAGATGAAGTTATCCATCAGGGAGT |
| amiC-out-F | GAGAGCGACTTTTTTATCA |
| amiC-out-R | GCGGGGTGGAGCAGC |
| amiC-in-F | ATGTCAGGATCCAACACT |
| amiC-in-R | TCATCCCCTTCTCGCCA |
| moaA up1 | TAAGTGAACTGCATGAATTCCCGGG GCAGAATTGGCATCAGGCTG |
| moaA up2 | AACCTCTTACGTGCCGATCAGTACACCTTTCCAGATACGG |
| moaA-chl-1 | CCGTATCTGGAAAGGTGTACTGATCGGCACGTAAGAGGTT |
| moaA-chl-2 | TGATCTCTCCTTTTGACGTTTTACGCCCCGCCCTGCCA |
| moaA down1 | AGTGGCAGGGCGGGGCGTAAAACGTCAAAAGGAGAGATCAGA |
| moaA down2 | GGCCCGATCCCAAGCTTCTTCTAGA AAGTCACCCGACTTGCCGC |
| moaA-out-F | TTACACGCTAGTATCGGCTTA |
| moaA-out-R | GTGATGGCCCGCTTCCTG |
| moaA-in-F | ATGGCTTCACAACTGACT |
| moaA-in-R | TTAGCCGCCAATGTACGA |
| cueO up1 | TAAGTGAACTGCATGAATTCCCGGG TACCAGCGTTATGGTTGGGTA |
| cueO up2 | AACCTCTTACGTGCCGATCAAGTTATTTCCTTATTCTTAAGCA |
| cueO-chl-1 | TTAAGAATAAGGAAATAACTTGATCGGCACGTAAGAGGTT |
| cueO-chl-2 | TATTTCCGAATACGGTCTTTTTACGCCCCGCCCTGCCA |
| cueO down1 | AGTGGCAGGGCGGGGCGTAAAAAGACCGTATTCGGAAATAT |
| cueO down2 | GGCCCGATCCCAAGCTTCTTCTAGAGTTAACGGTCAAAAGGTTCC |
| cueO-out-F | TTGCTTCTACCAGGGGCTTA |
| cueO-out-R | GCTACGCCAATGACCTATGA |
| cueO-in-F | TACCGATCCCTGATTTGCTC |
| cueO-in-R | CAGCACTTCGCTGACATTAC |
| yahJ up1 | TAAGTGAACTGCATGAATTCCCGGG CTCTCATAACCCCATCACCC |
| yahJ up2 | AACCTCTTACGTGCCGATCACTTACGTCCTTACGTTTTAATAAT |
| yahJ-chl-1 | TTAAAACGTAAGGACGTAAGTGATCGGCACGTAAGAGGTT |
| yahJ-chl-2 | GCAGGCGTTTCAAATTCGTATTACGCCCCGCCCTGCCA |
| yahJ down1 | AGTGGCAGGGCGGGGCGTAATACGA ATTTGAAACGCCTGCAA |
| yahJ down2 | GGCCCGATCCCAAGCTTCTTCTAGAAGCGAAGACACCATATTGATAA |
| yahJ-out-F | CCGCCTGGCAGCATGTT |
| yahJ-out-R | TCACTTTTGTGTATTTCTCTCA |
| yahJ-in-F | ATGAAAGAAAGCAATAGCCGC |
| yahJ-in-R | TCAACCTGCCACACTCCC |
| wcaM up1 | TAAGTGAACTGCATGAATTCCCGGGCAGACCTTACGCGGCATTCA |
| wcaM up2 | AACCTCTTACGTGCCGATCAGCGTTCTCCTCTATAAAGCCT |
| wcaM-chl-1 | GGCTTTATAGAGGAGAACGCTGATCGGCACGTAAGAGGTT |
| wcaM-chl-2 | GTTTATGAATGGTCGCAAATTTACGCCCCGCCCTGCCA |
| wcaM down1 | AGTGGCAGGGCGGGGCGTAAATTTGCGACCATTCATAAACTTA |
| wcaM down2 | GGCCCGATCCCAAGCTTCTTCTAGACACGCGCACAGAGCTGACA |
| wcaM-out-F | CGCAACGACTGGCGG |
| wcaM-out-R | TTTAGTCTGGTTGTTACGT |
| wcaM-in-F | ATGCCATTTAAAAAACTCTCCC |
| wcaM-in-R | CTACTCCCTCCGTTCCGG |
| mdoDup1 | TAAGTGAACTGCATGAATTCCCGGGGGCACCAGTCAGCAACGGT |
| mdoDup2 | AACCTCTTACGTGCCGATCAACCTGAAAGTGAGTCCTTCT |
| mdoD-chl-1 | AGAAGGACTCACTTTCAGGTTGATCGGCACGTAAGAGGTT |
| mdoD-chl-2 | AGGTACCGAAGAAAAAACGATTACGCCCCGCCCTGCCA |
| mdoDdown1 | AGTGGCAGGGCGGGGCGTAATCGTTTTTTCTTCGGTACCTT |
| mdoDdown2 | GGCCCGATCCCAAGCTTCTTCTAGACGAGGAGCGCATTACGCGA |
| mdoD-out-F | AATTGCCGATTAATGCATATT |
| mdoD-out-R | TCTGGAAACATACGCACT |
| mdoD-in-F | ATGGATCGTAGACGATTTATTAA |
| mdoD-in-R | TTAACTCATCACGCGGTCGT |
| ycbKup1 | TAAGTGAACTGCATGAATTCCCGGGAACGCGTGACTGGTTAAACGT |
| ycbKup2 | AACCTCTTACGTGCCGATCAGATAATCAAGTCTACAGGTCAA |
| ycbK-chl-1 | GACCTGTAGACTTGATTATCTGATCGGCACGTAAGAGGTT |
| ycbK-chl-2 | CCTGTTTCGTTAAGCGATTGTTACGCCCCGCCCTGCCA |
| ycbKdown1 | AGTGGCAGGGCGGGGCGTAACAATCGCTTAACGAAACAGG |
| ycbKdown2 | GGCCCGATCCCAAGCTTCTTCTAGAGATTTGGGAACAAGAGCTGA |
| ycbK-out-F | CTGACTGCCTTTGTTGGT |
| ycbK-out-R | CAGGCAAGCCTTGCAGC |
| ycbK-in-F | AAACTGCTGGCGCTTGGT |
| ycbK-in-R | GTATCAATATGCACAAAGTTA |
| efeOBup1 | TAAGTGAACTGCATGAATTCCCGGGATTTTATAAACATTCCGCTTGTAT |
| efeOBup2 | AACCTCTTACGTGCCGATCA GACGAGACTATCCCTTTAAAGT |
| efeOB-chl-1 | TTTAAAGGGATAGTCTCGTCTGATCGGCACGTAAGAGGTT |
| efeOB-chl-2 | ATCGGGCATATTTGCGTCGT TTACGCCCCGCCCTGCCA |
| efeOBdown1 | AGTGGCAGGGCGGGGCGTAAACGACGCAAATATGCCCGAT |
| efeOBdown2 | GGCCCGATCCCAAGCTTCTTCTAGAAATAGGGAGCAAACTTTTCTGC |
| efeOB-out-F | GAAGTCGCCGTCTGGT |
| efeOB-out-R | AAAAAAGCTGTGTGCTTTC |
| efeOB-in-F | TTACGGTTAACGCCGGGA |
| efeOB-in-R | AACATCATGCTCATGCTGCATT |
| fhuDup1 | TAAGTGAACTGCATGAATTCCCGGG GATCTGGCGCGAGTCGG |
| fhuDup2 | AACCTCTTACGTGCCGATCACAATAAACAAAACTCACAGGT |
| fhuD-chl-1 | CCTGTGAGTTTTGTTTATTGTGATCGGCACGTAAGAGGTT |
| fhuD-chl-2 | GAAAAGCGCAATTCGTTTACTTACGCCCCGCCCTGCCA |
| fhuDdown1 | AGTGGCAGGGCGGGGCGTAAGTAAACGAATTGCGCTTTTCC |
| fhuDdown2 | GGCCCGATCCCAAGCTTCTTCTAGATTCATATCCGGCGCGCTAAT |
| fhuD-out-F | TTATCTGGTCGCCCTGCG |
| fhuD-out-R | TCTGCTCGATGACGTCAATA |
| fhuD-in-F | ATGAGCGGCTTACCTCTT |
| fhuD-in-R | TCACGCTTTACCTCCGAT |
| fdnGup1 | AACTGCATGAATTCCCGGGAGAGCTCAGGAAAGGACGGGCGTAAA |
| fdnGup2 | CCAGTGATTTTTTTCTCCATTGCTTTCCTCTTTTTTCAGG |
| fdnG-chl-1 | CCTGAAAAAAGAGGAAAGCAATGGAGAAAAAAATCACTGGA |
| fdnG-chl-2 | ATCTGTTCGCCCCCTTACGCTTACGCCCCGCCCTGCCA |
| fdnGdown1 | AGTGGCAGGGCGGGGCGTAAGCGTAAGGGGGCGAACA |
| fdnGdown2 | GATCCCAAGCTTCTTCTAGAGGTACCGAAGAAGAACGAAATCCCG |
| fdnG-out-F | TTCCTCGCGCAGTAATACC |
| fdnG-out-R | AGTTAGTTGCGGACCTTTTGA |
| fdnG-in-F | CTGGATTACGTCAACAGCGA |
| fdnG-in-R | CCATACCCATGTTACCGAGC |
| fdoGup1 | AACTGCATGAATTCCCGGGAGAGCTCACCGCCGCCA GGATGAA |
| fdoGup2 | CGGGGCGTAAGTGTAACGGAGACGACATAT |
| fdoG-chl-1 | TCCG TTACACTTACGCCCCGCCCTGC |
| fdoG-chl-2 | GGGCTGTTGATGATCGGCACGTAAGAGG |
| fdoGdown1 | GTGCCGATCATCAACAGCCC ACAGCCTA |
| fdoGdown2 | GATCCCAAGCTTCTTCTAGAGGTACCATAAACCGTTGCCCGACC |
| fdoG-out-F | CGCGGAACGACGAATGATA |
| fdoG-out-R | GCGAATGTAAATGGGACGTG |
| fdoG-in-F | TTTCAGCGTGCGAATACGTT |
| fdoG-in-R | ATAGTTAACTCCGGTCGCTG |
| hybOA up1 | AACTGCATGAATTCCCGGGAGAGCTCGAATGCCACCAGCGAATAG |
| hybOA up2 | GTGGCAGGG CGGGGCGTAAACGACGGAGGAGACGATC |
| hybOA-chl-1 | ATGATCGTCTCCTCCGTCGTTTACGCCCCGCCCTGC |
| hybOA -chl-2 | GACTATCTCTCTGGAGTATCTGATCGGCACGTAAGAGGT |
| hybOA down1 | AACCTCTTACGTGCCGATCAGATACTCCAGAGAGATAGTC |
| hybOA down2 | GATCCCAAGCTTCTTCTAGAGGTACCTTTATGGTCGTCAGGCCC |
| hybOA-out-F | TACTGATGATTTTGCCGCCC |
| hybOA-out-R | CAGGTCTTCGCAACGGAATA |
| hybOA-in-F | TCTGACGCGGATAGTGGTAT |
| hybOA-in-R | CTGCAATTCTGCGATGTTGG |
| hyaAup1 | AACTGCATGAATTCCCGGGAGAGCTCAGAATTGGTCCGAGGATATAA |
| hyaAup2 | TTTTCTCCATATCGCACGTCTCTTCTCCT |
| hyaA-chl-1 | GACGTGCGATATGGAGAAAAAAATCACTGGA |
| hyaA-chl-2 | ATCCTCATTGTTACGCCCCGCCCTGC |
| hyaAdown1 | CGGGGCGTAACAATGAGGATAAACAGGCAT |
| hyaAdown2 | GATCCCAAGCTTCTTCTAGAGGTACCCCGTTAATCACCGCGCC |
| hyaA-out-F | CAAGGAGAAGAGACGTGCG |
| hyaA-out-R | GGCGTCCGGCATTATTGA |
| hyaA-in-F | GGATCCACGGTCTGGAATG |
| hyaA-in-R | GACGATCAAAGGTCACCATGT |
| nrfCup1 | AACTGCATGAATTCCCGGGAGAGCTCCACGCTCACT TCGAAGCG |
| nrfCup2 | TTTTCTCCATGGCTGCTCCTTAAGCAAC |
| nrfC-chl-1 | AGGAGCAGCCATGGAGAAAAAAATCACTGGA |
| nrfC-chl-2 | CTCCCCGTATTTACGCCCCGCCCTGC |
| nrfCdown1 | CGGGGCGTAAATACGGGGAG GTGAGCCA |
| nrfCdown2 | GATCCCAAGCTTCTTCTAGAGGTACCAACCGACTTCAGGAAGGAAT |
| nrfC-out-F | AATCCGAACTTTAACCCGGC |
| nrfC-out-R | AAGTCTGCGTCATTGGCTC |
| nrfC-in-F | GGTGGCGAAAACGTTGAATA |
| nrfC-in-R | TCCAGATTGCCAAACGTCAG |
| yagTup1 | AACTGCATGAATTCCCGGGAGAGCTCAACCTTCAGC TGATCGATC |
| yagTup2 | CGGGGCGTAATGCGGGGGAGATAAAATCAT |
| yagT-chl-1 | CTCCCCCGCATTACGCCCCGCCCTGC |
| yagT-chl-2 | GAGGCCTGGTATGGAGAAAAAAATCACTGGA |
| yagTdown1 | TTTTCTCCATACCAGGCCTCCGGTATTC |
| yagTdown2 | GATCCCAAGCTTCTTCTAGAGGTACCGATCTCAGGGTCTGGCTC |
| yagT-out-F | ACTCGTTCATAGGTAAACGCC |
| yagT-out-R | CCCTTTAGAATACCGGAGGC |
| yagT-in-F | ACAGATGTTGCCGCTCATAC |
| yagT-in-R | CCGCGCCCGAGATAATG |
| yghXup1 | AACTGCATGAATTCCCGGGAGAGCTCATAGAAGAAACTTCCTGATGC |
| yghXup2 | CGGGGCGTAAGATTAAAAAGGAAAATGTATGAA |
| yghX-chl-1 | CTTTTTAA TCTTACGCCCCGCCCTGC |
| yghX-chl-2 | TTTCAGGGAGATGGAGAAAAAAATCACTGGA |
| yghXdown1 | TTTTCTCCATCTCCCTGAAA TTTCAGGGG |
| yghXdown2 | GATCCCAAGCTTCTTCTAGAGGTACCTGGCGAAGATATGACTCAG |
| yghX-out-F | CCAACTGGCTCTGAAACTGT |
| yghX-out-R | ATGGTTCGAGGTAGAGCAGT |
| yghX-in-F | TCTTCGCGACCAAAGATCAA |
| yghX-in-R | TTAGCGAACACGAGGCAAG |
| napGup1 | AACTGCATGAATTCCCGGGAGAGCTCCTTTTGGCATCCGAATGGC |
| napGup2 | AGTGGCAGGGCGGGGCGTAACGGCTGGCTGGAGGGGAA |
| napG-chl-1 | TGTTCCCCTCCAGCCAGCCGTTACGCCCCGCCCTGCC |
| napG-chl-2 | TGGAGAAGGTGTAAGCCATTATGGAGAAAAAAATCACTGGAT |
| napGdown1 | CCAGTGATTTTTTTCTCCATAATGGCTTACACCTTCTCCA |
| napGdown2 | GATCCCAAGCTTCTTCTAGAGGTACCGCGCGATCCGCGCAACTT |
| napG-out-F | AGCCGAAGCGGTAATGGTGA |
| napG-out-R | AACCTCAAAATGGTCGCCGC |
| napG-in-F | CTGCGCCACCAGCCTTTTT |
| napG-in-R | GCGCGGTCAAACTGGAGAAG |
| ydhXup1 | AACTGCATGAATTCCCGGGAGAGCTCATTCGTGGGCGCGTAGCG |
| ydhXup2 | AGTGGCAGGGCGGGGCGTAAGCTATCACCGGCACTAACG |
| ydhX-chl-1 | TCGTTAGTGCCGGTGATAGCTTACGCCCCGCCCTGCC |
| ydhX-chl-2 | TCATACATTTTCCTTTTTAAATGGAGAAAAAAATCACTGGTA |
| ydhXdown1 | CCAGTGATTTTTTTCTCCATTCAAATGTTGACCGAACCGG |
| ydhXdown2 | GATCCCAAGCTTCTTCTAGAGGTACCCGCCATTGCACATGCCGT |
| ydhX-out-F | GGTGATAGCCATCGACCATGC |
| ydhX-out-R | ATGAACCCGTCGCAACATGC |
| ydhX-in-F | CTCGCAGGTCTGCTCGTGTA |
| ydhX-in-R | TATCGCCGGTTCGGTCAACA |
| pHSG-Apra-F | AATGAGACGTGGGTTCATGTGCAGCTCC |
| pHSG-Apra-R | AACTGCCTTATCATGAGCTCAGCCA |
| pHSG-pro-F | AAACGACGGCCAGTGAATTCTGTCGGTTGGCGCAAAACA |
| pHSG-pro-R | TGACGCCGACTGAGTGACATACATGTTCCTCTGTGGTAGAT |
| pHSG(Apra)-sufI-F | CTACCACAGA GGAACATGTATGTCACTCAGTCGGCGTCA |
| pHSG-(Apra)-sufI-R | GGGTCGACTCTAGAGGATCCTTACGGTACCGGGTTGACTAA |
| Apra-QE80-AF | TTCCCCGAAAAGTGCCACCTGACGTCATCGCATTCTTCGCATCCCG |
| Apra-QE80-AR | CATGATAATAATGGTTTCTTGACGTCCACCGACTATTTGCAACAGT |
| pQE80-GFP-Chl-Apra-F | ACGCCCGGTAGTGATCTTAT |
| pQE80-GFP-Chl-Apra-R | TGAAGACGAAAGGGCCTCGT |
